# Electronic evaluation of infection risk from room environment and prior room occupants: the transmission from room environment events (TREE) measure

**DOI:** 10.1017/ice.2024.194

**Published:** 2025-02

**Authors:** Clare Rock, Yea-Jen Hsu, Aaron M. Milstone, Alejandra B. Salinas, Lisa Maragakis, Nathan Kwon, Sara E. Cosgrove, Patricia J. Simner, Zunaira Virk, Abigail H. Vorsteg, Eili Klein, For the Centers for Disease Control and Prevention’s Prevention Epicenters and MInD Programs

**Affiliations:** 1 Division of Infectious Diseases, Johns Hopkins University School of Medicine, Baltimore, MD, USA; 2 The Johns Hopkins Hospital Department of Hospital Epidemiology and Infection Control, Baltimore, MD, USA; 3 Department of Health Policy and Management, Johns Hopkins University Bloomberg School of Public Health, Baltimore, MD, USA; 4 Division of Pediatric Infectious Diseases, Johns Hopkins University School of Medicine, Baltimore, MD, USA; 5 Division of Medical Microbiology, Johns Hopkins University School of Medicine, Baltimore, MD, USA; 6 Department of Emergency Medicine, Johns Hopkins University School of Medicine, Baltimore, MD, USA; 7 One Health Trust, Washington, DC, USA

## Abstract

Over a 2-year period, we identified Transmission from Room Environment Events (TREE) across the Johns Hopkins Health System, where the subsequent room occupant developed the same organism with the same antimicrobial susceptibilities as the patient who had previously occupied that room. Overall, the TREE rate was 50/100,000 inpatient days.

## Background

Hospital-acquired infections (HAIs) threaten patient safety, with increased associated mortality and hospital length of stay.^1–3^ The room environment plays an important role in pathogen transmission in the hospital. Patients have significantly increased independent odds of developing an infection with the same organism as the prior room occupant.^4,5^ However, despite the critical role of the room environment in pathogen transmission, it remains challenging to capture these transmissions. Additionally, some patients develop infections post-discharge, making it challenging to epidemiologically link to their hospitalization. While advances in whole genome sequencing (WGS) offer a potential for evaluating transmissions,^6^ WGS remains expensive, labor intensive, and is unlikely to be deployed in a wide-scale manner soon.

These challenges prevent the timely evaluation of environment-focused performance improvement interventions and limit the translation of novel interventions into real-world practice. An automated, consistent method for evaluating environmental transmissions over time and across hospitals using existing Electronic Health Record (EHR) data is needed. In this study, we evaluate spatiotemporal transmission events mediated via shared patient room environments. Specifically, we assess the ability to use routine information available in the EHR to calculate the Transmission from Room Environment Events (TREE) score, which measures the rate of all potential bacterial and fungal environmental transmissions in a hospital system over time. The intention is to use this novel scoring method to assess the hospital-level efforts to ensure a clean environment for patient care.

## Methods

We conducted an observational cohort study of all patients admitted to any of the five Johns Hopkins Health System (JHHS) academic and community hospitals with at least one positive microbiology result between January 2018 and February 2020 (see Table 1). All these hospitals have private rooms, apart from one of the academic hospitals where approximately half the rooms are shared. Routinely available EHR data on patient room occupation (e.g., unit/room number, transfer-into and transfer-out of room times), microbiology results, and demographics were bulk extracted. Hospital-onset infections (HOIs) were defined as a positive microbiology results for any organism collected as a clinical specimen (surveillance specimens were not included) at least 48 hours after hospital admission. Patients with more than one organism collected after 48 hours were counted as separate infections, but additional infections from the same organism were excluded (Figure S1). Potential transmission events were defined as an HOI in which (a) the prior occupant had an infection within 60 days of room occupancy. A sensitivity analysis using within 30 and 90 days of room occupancy was conducted to evaluate the impact of changing this time period on the measure. (This assumes the source patient remains infectious for the duration of the hospital admission after a positive result.). (b) The organism identified in exposed patient was collected within 180 days of exposure to the shared room environment, and (c) the organism identified in the exposed patient had identical antimicrobial susceptibility testing (AST) results as the organism identified in the prior occupant (e.g., sensitive, intermediate, resistant). Additional sensitivity analyses included events where AST results were identical at the minimum inhibitory concentration (MIC) level and a “surprise index” events where the phenotype of the organism identified at the MIC level was rare (<5%); we calculated the percentage of all isolates for each species that had the identical phenotype in the year prior to collection. Patient characteristics were described using frequencies and percentages, the overall aggregated rate of HOI per 100,000 patient days, and the TREE score (the rate of potential transmission events per 100,000 patient days). Results were calculated overall and aggregated at the hospital level, unit type level, and by organism. This study was approved by the Johns Hopkins Medicine Institutional Review Board.

**Table 1.** Characteristics of study patients

Patients	7,138
Encounters	8,049
Age, years	
0–18	508 (7.1)
19–64	3,471 (48.6)
65+	3,159 (44.3)
Gender	
Female	3,494 (48.9)
Male	3,642 (51.0)
Nonbinary/Other/Unknown	2 (0.0)
Race	
Black or African American	2,304 (32.3)
Asian	191 (2.7)
American Indian or Alaskan Native	17 (0.2)
Native Hawaiian or Other Pacific Islander	1 (0.0)
White	3,997 (56.0)
Other/Unknown/Some Other Race	531 (7.4)
Ethnicity	
Hispanic	234 (3.3)
Not Hispanic	6,713 (94.0)
Unknown	82 (1.1)
No. of hospital admissions	
One	6,460 (90.5)
Two	514 (7.2)
Three	115 (1.6)
Four	49 (0.7)

## Results

Over the study period, there were 2,903,509 patient exposure days, and 7,138 unique patients admitted to the JHHS with a HOI identified (Table 1). These patients had 12,717 HOIs, of which 1,453 had evidence of exposure from a prior room occupant in the past 60 days for a rate of potential transmission events (TREE) score of 50/100,000 inpatient days (Table 2). Potential exposures and the rate of infection were higher in the academic hospitals compared to the community hospitals. Intensive care units had more than three times the TREE rate of regular floor units. The percentage of HOIs that were associated with prior room occupants was relatively stable over time (Figure S2).

**Table 2.** Incidents of hospital-onset infections and percent of patients exposed to infected prior room occupants overall and by hospital and unit type and organism

	Patient days	Number and rate per 100,000 inpatient days of hospital onset infections	Number and rate per 100,000 inpatient days of infections with exposure from prior room occupants^**^		
			30 days until room exposure	60 days until room exposure	90 days until room exposure
All	2,903,509	12,717 (438.0)	895 (30.8)	1453 (50.0)	1850 (63.7)
By hospital					
Academic hospital #1	1,292,499	7,557 (584.7)	484 (37.4)	779 (60.3)	979 (75.7)
Academic hospital #2	467,802	2,532 (541.3)	233 (49.8)	382 (81.7)	489 (104.5)
Community hospital #1	484,252	1,175 (242.6)	101 (20.9)	160 (33.0)	205 (42.3)
Community hospital #2	332,335	1,030 (309.9)	45 (13.5)	77 (23.2)	108 (32.5)
Community hospital #3	326,621	582 (178.2)	32 (9.8)	55 (16.8)	69 (21.1)
By unit type					
Inpatient ICU	345,470	4,381 (1,268.1)	364 (105.4)	564 (163.3)	711 (205.8)
Inpatient Floor	1,353,830	6,415 (473.8)	407 (30.1)	666 (49.2)	852 (62.9)
Other^*^	1,204,209	2080 (172.7)	124 (10.3)	223 (18.5)	287 (23.8)
By Organism					
*Escherichia coli*	2,903,509	1792 (61.7)	102 (3.5)	197 (6.8)	245 (8.4)
*Staphylococcus aureus*	2,903,509	1744 (60.1)	227 (7.8)	360 (12.4)	453 (15.6)
MSSA	2,903,509	970 (33.4)	155 (5.3)	250 (8.6)	299 (10.3)
MRSA	2,903,509	804 (27.7)	72 (2.5)	110 (3.8)	154 (5.3)
*Enterococcus faecalis/faecium*	2,903,509	1566 (53.9)	93 (3.2)	158 (5.4)	191 (6.6)
VSE	2,903,509	1262 (43.5)	75 (2.6)	127 (4.4)	148 (5.1)
VRE	2,903,509	345 (11.9)	18 (0.6)	31 (1.1)	43 (1.5)
*Pseudomonas aeruginosa*	2,903,509	1284 (44.2)	299 (10.3)	433 (14.9)	549 (18.9)
*Klebsiella pneumoniae*	2,903,509	1089 (37.5)	68 (2.3)	119 (4.1)	163 (5.6)
*Staphylococcus (CONS)*	2,903,509	774 (26.7)	29 (1.0)	56 (1.9)	80 (2.8)
*Enterobacter cloacae*	2,903,509	518 (17.8)	19 (0.7)	31 (1.1)	43 (1.5)
*Proteus mirabilis*	2,903,509	456 (15.7)	15 (0.5)	30 (1.0)	42 (1.4)
*Stenotrophomonas maltophilia*	2,903,509	281 (9.7)	0 (0.0)	0 (0.0)	0 (0.0)
*Serratia marcescens*	2,903,509	264 (9.1)	20 (0.7)	38 (1.3)	47 (1.6)
*Klebsiella oxytoca*	2,903,509	223 (7.7)	6 (0.2)	7 (0.2)	11 (0.4)
*Corynebacterium striatum*	2,903,509	174 (6.0)	3 (0.1)	6 (0.2)	9 (0.3)
Other	2,903,509	168 (5.8)	3 (0.1)	8 (0.3)	10 (0.3)

* Including procedural units and emergency departments.

** This looks back 30, 60, & 90 days from date/time that a patient enters a room to determine if any prior occupant was positive for an organism, and looks out 180 days after end of exposure (e.g., left that room) for positive infections in patient; MRSA = Methicillin-resistant *S. aureus*; MSSA = Methicillin-sensitive *S. aureus*; VSE = Vancomycin-susceptible enterococci; VRE = Vancomycin-resistant enterococci.

We explored different parameters in this measure development (see Table S1). The number of TREE events increased with a longer lookback period, but the increase diminished the longer the lookback (Table 2). Using MIC or the surprise index significantly reduced the number of TREE events but increased the likelihood that they were true transmission events (Table S2). While the majority of infections were excluded under the surprise index sensitivity analysis at 5%, because of the long tail of the index, a similar result would be found at 10% (Figure S2). Finally, reducing the time period after exposure end had only a marginal impact on the number of TREE events (Table S3).

## Discussion

We present a novel method, TREE (Transmission from Room Environment Events (TREE), which scores the potential impact of hospital environments on bacterial and fungal pathogen transmission. The focus of this paper was the development of this novel measure, with many of the parameters undergoing sensitivity analyses to elucidate the best fit. We found that using 60 days as the time frame to look back at all patients that occupied the room seemed to be optimal. There was marked increase in TREE measure from 30 to 60 days (895–1,453). However, there was a much smaller relative increase from 60 to 90 days (1,453–1,850), indicating that there may not be as many TREE events occurring during that 60–90-day time frame. We also used a “surprise index,” with the aim of trying to improve the accuracy of the measure by evaluating rare organisms and susceptibility testing results, as those rare combinations meeting TREE definition may have a higher probability of being actual environmental transmission events.

Overall, applying this scoring method to our hospital system found a rate of 50 potential environmental-related transmission events per 100,000 inpatient days. We anticipate the actual TREE rate may be higher, as the current measure relies on clinical culture positivity alone and does not capture transmission resulting in asymptomatic colonization. TREE events, using 30 days as the length of time the prior occupant had an infection and 180 post-exposure detection window, represented ∼6.6% of HOIs. Prior studies evaluating single multidrug resistant organisms (MDROs) have found an increased likelihood of acquisition when the prior occupant was positive.^4,5^ However, we aggregated all bacterial and fungal infections and expressed this as an event rate of overall microbiology results as a novel metric, making direct comparison challenging.^7,8^

Patient-related factors likely influence the TREE rate, as we found patients in the ICU at higher risk of infection. As academic hospitals tend to treat a sicker population with greater ICU use, the relatively higher risk of ICU patients which may explain the difference noted between academic and community hospitals. Thus, the expansion of this type of score to other hospitals should be adequately risk-adjusted, like other measures reported by CDC-NHSN.

This study highlights the benefits of using the EHR to evaluate potential environmental transmission events at a large scale. A limitation of the study includes lack of WGS to confirm transmission events. While WGS would provide greater certainty of transmission, currently it is not feasible to implement for monitoring and evaluating ongoing transmissions. This novel outcome, which uses regularly collected data from the EHR, may be used as an ongoing measure of pathogen transmission in the healthcare environment, likely including from prior patient occupant and from room environment e.g., water sources. Additionally, this outcome may help identify units and hospitals with significant opportunities for improvement in environmental cleaning and for evaluating the response to interventions successful at interrupting this specific pathway. Next steps of this work, in addition to further evaluation and validation, include using the same large data set in the EHR to simultaneously evaluate TREE and healthcare worker and patient connections to understand the potential role of these different transmission pathways. We also plan to compare individual hospital units in a risk-adjusted method to evaluate which hospital units may have higher than expected TREE rates and may benefit from a targeted quality improvement environmental intervention.

## Supporting information

Rock et al. supplementary material Rock et al. supplementary material

## Supplementary material

The supplementary material for this article can be found at https://doi.org/10.1017/ice.2024.194.
