# Supplementary material for: Electronic evaluation of infection risk from room environment and prior room occupants: the transmission from room environment events (TREE) measure
Source: Infect Control Hosp Epidemiol. 2024 Dec 12;46(2):209–12. doi: 10.1017/ice.2024.194 (PMC11790327; doi:10.1017/ice.2024.194)
Supplement: Rock et al. supplementary material [file S0899823X24001946sup001.docx]

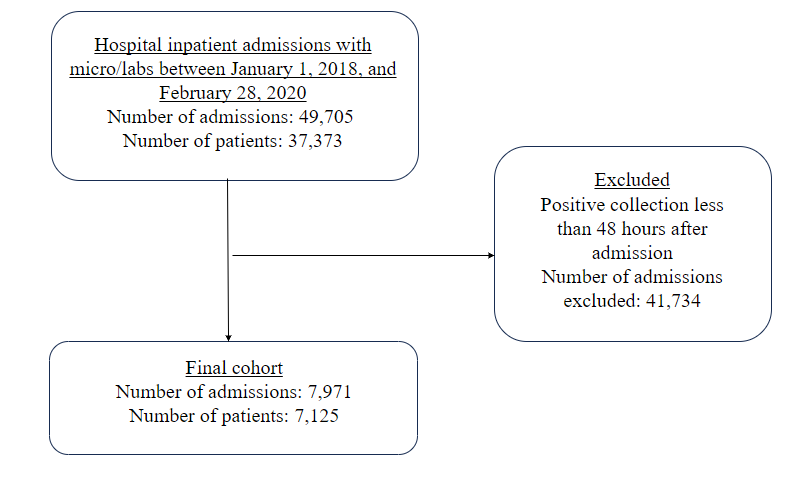


**Supplementary Figure 1. Flowchart demonstrating patients included in the cohort.**

* Included organisms: *Staphylococcus, Escherichia, Pseudomonas, Enterococcus, Klebsiella, Streptococcus, Proteus, Enterobacter, Haemophilus, Serratia, Citrobacter, Stenotrophomonas, Acinetobacter, Corynebacterium, Candida, Morganella, Achromobacter, Providencia, Salmonella, Mycobacterium, Burkholderia, Bacteroides, Alcaligenes, Propionibacterium, Clostridium.*


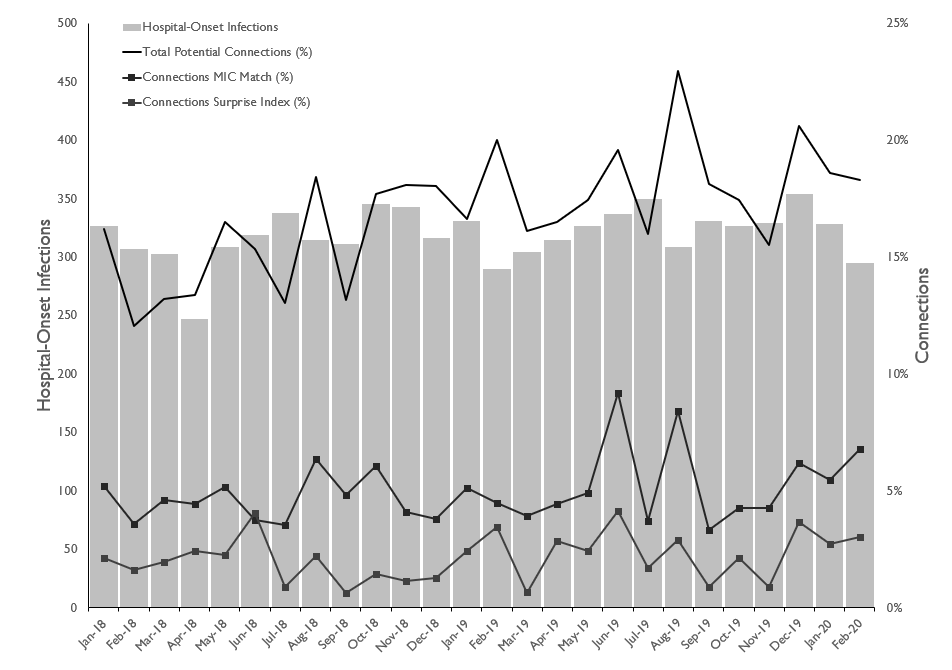
**Supplementary Figure 2. Trends in hospital-onset infections**

Number of hospital-onset infections in total frequency per month (gray bars that correspond to left y-axis) and the percentage of infections that had a potential room exposure (black lines that correspond to the percentage of the total observed connections on the right y-axis) using 60 days until room exposure. The MIC match assumes further phenotypic matching at both the AST level as well as the MIC level, which is a conservative estimate given the variability in the doubling dilutions. The surprise index measures the relative rarity of MIC-matched phenotypes.


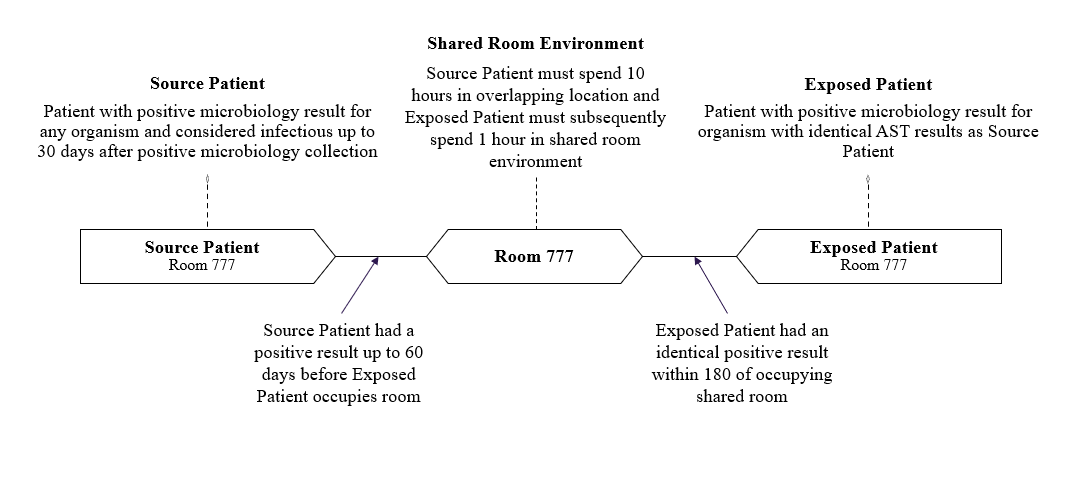


**Supplementary Figure 3. Definition of a transmission event**

This figure illustrates the definition of a transmission event used for the development of this measure which is described in the Methods.

| **Supplementary Table 1. Sensitivity analysis of incidents of hospital onset infections and percentage of patients exposed to infected prior room occupants overall and by hospital and unit type and organism.** | | | | | | |
| --- | --- | --- | --- | --- | --- | --- |
|  | Number of infections with exposure from prior room occupants (MIC match) | | | Number of infections with exposure from prior room occupants (Organism Surprise Index <.05)† | | |
|  | 30 days until room exposure | 60 days until room exposure | 90 days until room exposure | 30 days until room exposure | 60 days until room exposure | 90 days until room exposure |
| All | 257 (8.9) | 437 (15.1) | 554 (19.1) | 103 (3.5) | 184 (6.3) | 250 (8.6) |
| By hospital |  |  |  |  |  |  |
| Academic hospital #1 | 108 (8.4) | 187 (14.5) | 236 (18.3) | 28 (2.2) | 54 (4.2) | 70 (5.4) |
| Academic hospital #2 | 61 (13.0) | 96 (20.5) | 120 (25.7) | 12 (2.6) | 25 (5.3) | 37 (7.9) |
| Community hospital #1 | 61 (12.6) | 103 (21.3) | 137 (28.3) | 41 (8.5) | 67 (13.8) | 90 (18.6) |
| Community hospital #2 | 10 (3.0) | 17 (5.1) | 19 (5.7) | 0 (0.0) | 2 (0.6) | 6 (1.8) |
| Community hospital #3 | 17 (5.2) | 34 (10.4) | 42 (12.9) | 22 (6.7) | 36 (11.0) | 47 (14.4) |
| By unit type |  |  |  |  |  |  |
| Inpatient ICU | 106 (30.7) | 172 (49.8) | 215 (62.2) | 38 (11.0) | 74 (21.4) | 102 (29.5) |
| Inpatient Floor | 112 (8.3) | 185 (13.7) | 242 (17.9) | 52 (3.8) | 87 (6.4) | 110 (8.1) |
| Other* | 39 (3.2) | 80 (6.6) | 97 (8.1) | 13 (1.1) | 23 (1.9) | 38 (3.2) |
| By Organism |  |  |  |  |  |  |
| *Escherichia coli* | 44 (1.5) | 82 (2.8) | 106 (3.7) | 25 (0.9) | 51 (1.8) | 64 (2.2) |
| *Staphylococcus aureus* | 102 (3.5) | 156 (5.4) | 191 (6.6) | 19 (0.7) | 34 (1.2) | 53 (1.8) |
| MSSA | 69 (2.4) | 104 (3.6) | 118 (4.1) | 9 (0.3) | 19 (0.7) | 29 (1.0) |
| MRSA | 33 (1.1) | 52 (1.8) | 73 (2.5) | 10 (0.3) | 15 (0.5) | 24 (0.8) |
| *Enterococcus faecalis/faecium* | 25 (0.9) | 46 (1.6) | 59 (2.0) | 11 (0.4) | 20 (0.7) | 20 (0.7) |
| VSE | 23 (0.8) | 42 (1.4) | 50 (1.7) | 10 (0.3) | 19 (0.7) | 19 (0.7) |
| VRE | 2 (0.1) | 4 (0.1) | 9 (0.3) | 1 (0.0) | 1 (0.0) | 1 (0.0) |
| *Pseudomonas aeruginosa* | 34 (1.2) | 57 (2.0) | 75 (2.6) | 26 (0.9) | 40 (1.4) | 56 (1.9) |
| *Klebsiella pneumoniae* | 20 (0.7) | 34 (1.2) | 47 (1.6) | 9 (0.3) | 20 (0.7) | 29 (1.0) |
| *Staphylococcus (CONS)* | 8 (0.3) | 17 (0.6) | 19 (0.7) | 2 (0.1) | 5 (0.2) | 13 (0.4) |
| *Enterobacter cloacae* | 7 (0.2) | 12 (0.4) | 16 (0.6) | 4 (0.1) | 6 (0.2) | 8 (0.3) |
| *Proteus mirabilis* | 7 (0.2) | 14 (0.5) | 18 (0.6) | 1 (0.0) | 1 (0.0) | 1 (0.0) |
| *Stenotrophomonas maltophilia* | 0 (0.0) | 0 (0.0) | 0 (0.0) | 0 (0.0) | 0 (0.0) | 0 (0.0) |
| *Serratia marcescens* | 5 (0.2) | 9 (0.3) | 13 (0.4) | 2 (0.1) | 3 (0.1) | 3 (0.1) |
| *Klebsiella oxytoca* | 1 (0.0) | 1 (0.0) | 1 (0.0) | 2 (0.1) | 3 (0.1) | 3 (0.1) |
| *Corynebacterium striatum* | 0 (0.0) | 1 (0.0) | 1 (0.0) | 0 (0.0) | 0 (0.0) | 0 (0.0) |
| Other | 0 (0.0) | 2 (0.1) | 3 (0.1) | 0 (0.0) | 0 (0.0) | 0 (0.0) |
| *Including procedural units and emergency departments; ** assumes exposure location not where specimen taken; † measures the relative rarity of each organism phenotype based on AST and MIC levels, lower percentages means rarer phenotype. MRSA = Methicillin-resistant *S. aureus*; MSSA = Methicillin-sensitive *S. aureus*; VSE = Vancomycin-susceptible enterococci; VRE = Vancomycin-resistant enterococci | | | | | | |

| **Supplementary Table 2. Sensitivity analysis of patients exposed to infected prior room occupants overall and by hospital and unit type for post exposure surveillance time** | | | | |
| --- | --- | --- | --- | --- |
|  | 7 days | 30 days | 60 days | 90 days |
| All | 959 | 1,371 | 1,436 | 1,444 |
| By hospital |  |  |  |  |
| Academic hospital #1 | 473 | 711 | 762 | 767 |
| Academic hospital #2 | 256 | 374 | 382 | 385 |
| Community hospital #1 | 113 | 154 | 159 | 159 |
| Community hospital #2 | 71 | 78 | 78 | 78 |
| Community hospital #3 | 46 | 54 | 55 | 55 |
| By unit type |  |  |  |  |
| Inpatient ICU | 422 | 624 | 658 | 663 |
| Inpatient Floor | 359 | 529 | 554 | 556 |
| Other* | 178 | 218 | 224 | 225 |
| By Organism |  |  |  |  |
| *Escherichia coli* | 144 | 194 | 197 | 197 |
| *Staphylococcus aureus* | 268 | 348 | 358 | 358 |
| MSSA | 187 | 242 | 248 | 248 |
| MRSA | 81 | 106 | 110 | 110 |
| *Enterococcus faecalis/faecium* | 92 | 138 | 150 | 155 |
| VSE | 78 | 113 | 124 | 127 |
| VRE | 14 | 25 | 26 | 28 |
| *Pseudomonas aeruginosa* | 274 | 399 | 427 | 428 |
| *Klebsiella pneumoniae* | 75 | 114 | 119 | 119 |
| *Staphylococcus (CONS)* | 34 | 53 | 56 | 56 |
| *Enterobacter cloacae* | 16 | 28 | 31 | 31 |
| *Proteus mirabilis* | 14 | 29 | 29 | 31 |
| *Stenotrophomonas maltophilia* | 0 | 0 | 0 | 0 |
| *Serratia marcescens* | 23 | 35 | 36 | 36 |
| *Klebsiella oxytoca* | 2 | 6 | 6 | 6 |
| *Corynebacterium striatum* | 4 | 6 | 6 | 6 |
| Other | 17 | 28 | 29 | 29 |
| *Including procedural units and emergency departments; cases based on days after exposure ends until surveillance for new infection concludes. MRSA = Methicillin-resistant *S. aureus*; MSSA = Methicillin-sensitive *S. aureus*; VSE = Vancomycin-susceptible enterococci; VRE = Vancomycin-resistant enterococci | | | | |

| **Supplementary Table 3. Multidrug-resistant organism (MDRO) definitions** | |
| --- | --- |
| Type | Definition |
| Methicillin-resistant *Staphylococcus aureus ^1^* | Includes *S. aureus* cultured from any specimen that tests oxacillin-resistant, ceoxitin-resistant, or methicillin-resistant by standard susceptibility testing methods, or by a laboratory test that is FDA-approved for MRSA detection from isolated colonies; these methods may also include a positive result by any FDA-approved test for MRSA detection from specific sources |
| Vancomycin-resistant *Enterococcus* spp. ^1^ | Any *Enterococcus* spp. (regardless of whether identified to the species level), that is resistant to vancomycin, by standard susceptibility testing methods or by results from any FDA-approved test for VRE detection from specific specimen sources. |
| CRE ^2^ | Any *Enterobacteriaceae* spp. testing resistant to any carbapenem including doripenem, ertapenem, imipenem or meromenem using the current CLSI breakpoints; or by a positive result for any method FDA approved for carapenemase detection. |
| MDR-*Acinetobacter ^1^* | Non-susceptibility (i.e., resistant or intermediate) to at least one agent in at least 3 antimicrobial classes of the following 6 classes:   - Ampicillin/sulbactam - Cephalosporins (cefepime, ceftazidime) - β-lactam/β-lactam β-lactamase inhibitor combination (piperacillin, piperacillin/tazobactam) - Carbapenems (imipenem,meropenem, doripenem) - Fluoroquinolones (ciprofloxacin or levofloxacin) - Aminoglycosides (gentamicin, tobramycin, or amikacin) |
| MDR-*Pseudomonas ^3^* | Non-susceptibility (i.e., resistant or intermediate) to at least one agent in at least 3 antimicrobial classes of the following 5 classes:   - Cephalosporins (cefepime, ceftazidime) - β-lactam/β-lactam β-lactamase inhibitor combination (piperacillin, piperacillin/tazobactam) - Carbapenems (imipenem, meropenem, doripenem) - Fluoroquinolones (ciprofloxacin or levofloxacin) - Aminoglycosides (gentamicin, tobramycin, or amikacin) |
| Extended-spectrum beta-lactamase Gram negatives ^1^ | Enterobacteriaceae spp. non-susceptible (i.e., resistant or intermediate) to ceftazidime, cefepime, ceftriaxone, or cefotaxime.  Pseudomonas aeruginosa non-susceptible (i.e., resistant or intermediate) to ceftazidime or cefepime. |
| *Clostridium difficile ^1^* | A positive laboratory test result for C. difficile toxin A or B, (includes molecular assays [PCR] or toxin assays)  OR  A toxin-producing C. difficile organism detected by culture or other laboratory means performed on a stool sample. |
| Drug-resistant *Streptococcus pneumoniae ^1^* | *S. pneumoniae* isolated from a sterile site and nonsusceptible to "at least one antimicrobial agent currently approved for use in treating pneumococcal infection." (NO LONGER INCLUDED IN LIST) |
| 1. CDC. Multidrug-Resistant Organism & Clostridium difficile Infection (MDRO/CDI) Module. January 2014. <http://www.cdc.gov/nhsn/PDFs/pscManual/12pscMDRO_CDADcurrent.pdf> 2. Oregon Public Health Division, OAR 333-019-0015 3. Sievert DM et al. Antimicrobial-Resistant Pathogens Associated with Healthcare-Associated Infections: Summary of Data Reported to the National Healthcare Safety Network at the Centers for Disease Control and Prevention, 2009–2010. ICHE 2013;34:1–14. | |
